# Development of a Resource for Health Professionals to Raise Advance Care Planning Topics During Kidney Care Consultations: A Multiple User-Centered Design

**DOI:** 10.1016/j.xkme.2024.100874

**Published:** 2024-07-18

**Authors:** Anna Winterbottom, Helen Hurst, Fliss E.M. Murtagh, Hilary L. Bekker, Paula Ormandy, Barnaby Hole, Lynne Russon, Emma Murphy, Keith Bucknall, Andrew Mooney

**Affiliations:** 1 Leeds Renal Unit, Leeds Teaching Hospitals NHS Trust & Institute of Health Sciences, School of Medicine, University of Leeds, Leeds, UK; 2 School of Health and Society, University of Salford and Northern Care Alliance NHS Foundation Trust, Salford, UK; 3 Wolfson Palliative Care Research Centre, Hull York Medical School, University of Hull, Hull, UK; 4 Institute of Health Sciences, School of Medicine, University of Leeds, Leeds, UK; 5 School of Health and Society, University of Salford, Salford, UK; 6 Population Health Sciences, Bristol Medical School, University of Bristol, Bristol, UK and North Bristol NHS Foundation Trust, Southmead Hospital, Bristol, UK; 7 Leeds Renal Unit, Leeds Teaching Hospitals NHS Trust and Wheatfields Hospice, Leeds, UK; 8 University Hospitals Coventry and Warwickshire NHS Trust, Centre for Care Excellence, and Coventry University, Coventry, UK; 9 Patient and Public Involvement chair - lay member; kidney transplant recipient; 10 Leeds Renal Unit, Leeds Teaching Hospitals NHS Trust, Leeds, UK

**Keywords:** Advance care planning (personalized care planning, anticipatory care), end-of-life care, health care professionals (renal physicians, clinicians, kidney doctors, nephrologists, dialysis nurses, renal nurses, kidney nurses, nephrology nurses), kidney failure (advanced kidney disease, end-stage kidney failure, end-stage kidney disease, chronic kidney disease), palliative care (palliative treatment, hospice care)

## Abstract

**Rationale & Objective:**

Planning and delivering treatment pathways that integrate end-of-life care, frailty assessment, and enhanced supportive care is a service priority. Despite this, people with kidney failure are less likely to have an advance care plan and receive hospice and palliative care compared with other chronic illness populations. This is linked to health professionals feeling unskilled initiating conversations around future treatment and care options. This article describes research underpinning the development of a guide for kidney health professionals discussing end-of-life and advance care planning options with people with kidney failure and family members.

**Study Design:**

The study comprised 2 parts: an initial cross-sectional qualitative approach using in-depth interviews with older adults with kidney failure and (bereaved) carers followed by resource development with input from multiple stakeholders.

**Setting & Participants:**

Older adults with kidney failure and (bereaved) carers recruited from 2 renal units in the North of England and by online advertisements with national United Kingdom-based kidney patient charities. Resource development included input from co-applicants, independent advisory committee, patient and public involvement team, multidisciplinary health professionals and academics in the United Kingdom and Denmark.

**Analytical Approach:**

Thematic analysis was used to analyze the data.

**Results:**

Twenty-seven people were interviewed: older adults with kidney failure (n = 18), carers (n = 5), bereaved carers (n = 4). Five themes are described: the context within which end-of-life conversations take place, preferences for end-of-life treatment and care, family members’ role and needs in supporting people with kidney failure at the end-of-life, expectations and experience of dialysis treatment, and beliefs and experiences of death and dying.

**Limitations:**

Participants were mainly White, British, and receiving hemodialysis.

**Conclusions:**

People with (lived) experience of kidney failure informed a guide which aims to build on health professionals existing skills and improve confidence having conversations about future treatment and care. Kidney teams have expressed interest implementing the guide in practice and within their broader communications training packages.

## Plain-Language Summary

Delivering treatment pathways integrating end-of-life care, frailty assessment, and enhanced supportive care is a service priority. Despite this, people with kidney failure are less likely to have an advance care plan and receive hospice and palliative care compared with other chronic illness populations. This article describes how people with (lived) experience of kidney failure informed a guide to build on health professionals existing skills and improve confidence having conversations about future treatment and care. The study comprised 2 parts: cross-sectional qualitative approach using in-depth interviews with older adults with kidney failure and (bereaved) carers followed by resource development with input from coapplicants, an independent advisory committee, a patient and public involvement team, multidisciplinary health professionals, and academics.

It is internationally recognized that kidney failure affects an increasingly old, frail and multi-morbid population.^1^ Many agencies highlight the importance of integrating end-of-life care pathways, frailty assessment, and enhanced supportive care to this group.^2^ Despite this, linked to a lack of initiation of such discussions by health care professionals, people with kidney failure are less likely to have an advance care plan or receive hospice and palliative care compared with other chronic illness populations. People with kidney failure experience high rates of hospitalization and critical care unit admissions and other intensive treatments in the last months of life.^3^

Kidney health professionals report feeling unprepared to talk about end-of-life issues for several reasons, including fear of upsetting patients, lack of time/training, and feeling unconfident in their skills.^4^ People with kidney failure may have unrealistic expectations about the potential benefits of treatment,^5^ overestimate their prognosis, and experience regret at starting dialysis.^6^ Without health care professional support, it is unlikely people with kidney failure will be able to express their preferences about future care or participate in discussions about changes in treatment when the benefits of treatments are unclear.^7^

Patient decision aids have been developed to support people with kidney failure to be proactively involved in kidney management decisions,^8^ and their effectiveness may be enhanced with decision-coaching.^9^ Enhancing clinicians’ skills and confidence to talk to their patients about what might be important to them for the next phase of disease management, and being confident to elicit what might be important to them about this change in health state, is essential to agreeing and implement future care plans.^8^ A realist synthesis of advance care planning interventions provides mixed evidence for their effectiveness, highlighting the importance of staff training aimed at instilling staff with the confidence to start conversations, and emphasizing the importance of holding them.^10^ Other specialties, using the lived experience of people with heart failure, dementia, and young people with life-limiting conditions, developed resources supporting health professionals to engage in these difficult conversations.^11^, ^12^, ^13^ This article describes the research underpinning the development of a similar resource for use in renal practice. It aims to support a systematic and effective way of improving communication, planning, and decision making among people with kidney failure, their families, and kidney health professionals.^14^ The study objectives are as follows:

1) Understand the experiences, views, and preferences for end-of-life kidney treatment and care and how this is communicated to people with kidney failure and their family members.
2) Co-design an evidence-based resource for kidney health professionals to support their skills and confidence in engaging in conversations, planning, and decision making around a people with kidney failure patient preferences for future treatment and end-of-life care.

## Methods

### Study Design

The study comprised 2 parts: 1) cross-sectional qualitative in-depth interviews with older adults with kidney failure and/or their carers (all carers in the sample were the spouse or children of people with kidney failure and are herein referred to as family members); 2) iterative co-design of a prototype conversation guide. The study builds on previous research to develop codesigned interventions supporting people with kidney failure to engage in shared decision making about dialysis, conservative management, and transplant options with their kidney health professionals.^15^^,^^16^ Our research methods are guided by the Medical Research Council complex intervention development and evaluation framework; our interventions are guided with reference to a multiple decision maker theoretical framework, MIND-IT.^8^

### Patient and Public Involvement (PPI) Advisory Team

Co-author (KB) led a PPI advisory group with 2 additional members and supported by co-authors (AW and PO). PPI members provided input to all stages of the project. KB represented PPI members at 2 steering group meetings.

### Independent Advisory Group

An independent advisory group was convened and comprised kidney and palliative care professionals, a decision scientist, and a person with kidney failure. The group met with co-applicants twice during the project providing oversight of the development of the research, feedback on resource development, and dissemination activities.

### Part 1: In-Depth Interviews

#### Setting

Two renal units in the North of England, Leeds Teaching Hospitals NHS Trust and Manchester University Hospital NHS Foundation Trust, and United Kingdom-based participants recruited by online advertisements with 2 national, kidney patient charities, namely, Kidney Patient Involvement Network (KPIN) and National Kidney Federation (NKF).

#### Sample

Participants were purposively sampled to ensure recruitment of 3 groups: people with kidney failure with a range of treatment experiences, family members, and bereaved family members. People with kidney failure were eligible if they were aged over 70 years and receiving dialysis or of any age and had chosen conservative kidney management. To enrich experiences of discussions about patient and future deterioration in health, we purposively oversampled for people who (1) were identified by the clinical team as having progressive frailty, (2) were receiving a reduced dialysis prescription to relieve treatment burden, (3) had been diagnosed with another life-limiting illness, (4) had received dialysis for 5+ years and were not on the transplant list, and (5) had been reviewed by a health care of the elderly consultant before starting dialysis (Leeds only). Family members of those meeting these inclusion criteria were included. Bereaved family members of people with kidney failure who were 3 months+ post bereavement were also invited to interview.

### Materials

Study information sheets and consent forms were developed to inform and recruit participants. An interview guide was developed and piloted with the PPI advisory group (Appendix S1). Open-ended questions explored how people with kidney failure and family members make sense of living with kidney failure toward the end-of-life, views, preferences and experiences for end-of-life kidney care, and the role of family members.

### Recruitment

Kidney health care professionals, including predialysis nurses, hemodialysis nurses, a consultant nurse, identified people with kidney failure meeting the inclusion criteria. In addition, interested parties responded to an online advertisement through 2 patient charities. A cover letter, consent form, and patient information sheet was sent by the post to eligible participants. Those people wishing to take part contacted the study lead (AW/HH) to discuss the study, answer questions, organize a suitable time for the interview to take place, and go through the written consent procedure. Consent forms were posted back to the study lead before the interview. Interviews were continued until it was felt that no new data were being generated.^17^ Study advertisements were placed on Kidney Patient Involvement Network website and National Kidney Federation newsletter. Participants were given the opportunity to receive a copy of the resource once it had been developed.

### Data Collection

Interviews were conducted by AW, a senior health services researcher, and HH, a consultant nurse and professor of nursing with a clinical and academic background, who were not known to the participants, not involved in their care, and were introduced as a researcher. Interviews took place at the participants preferred location, using Zoom, on the telephone, or in the person’s home and lasted no longer than 1 hour. Coronavirus disease (COVID)-19 restrictions affected the interview location, with more being conducted remotely than originally anticipated. Given the sensitive topic area, steps were taken to ensure that distress was minimized during recruitment and interview.^18^ Audio recordings were transcribed verbatim, anonymized by a third party, and pseudonyms assigned for reporting the findings.

### Analysis

NVivo software (release 1.6.1, QSR International, 2022) managed the data, which were analyzed using thematic analysis.^19^ Analysis was conducted using a critical realist approach, whereby it is acknowledged that an external reality exists that is knowable and that people’s experiences are subjective. Analysis involved a 5-step process, including, familiarization with the data, generation of initial codes, searching for themes, refining and reviewing themes, and defining and labeling themes. Emerging themes were discussed with team members to help identify potential errors, biases, and oversights. Consolidated criteria for Reporting Qualitative studies (COREQ) were followed.^20^

### Part 2: Resource Development

The resource is a reference book designed to address a specific service need identified by kidney health professionals around improving skills in engaging in conversations about kidney care management that may be challenging. The guide was produced using a framework for multiple stakeholder complex interventions,^8^ structured communication guides based on person-centered communication,^21^^,^^22^ and using a user-centered design approach. Iterative drafts were produced by drawing on the following resources: interview findings, resources supporting end-of-life discussions for health professionals,^11^, ^12^, ^13^^,^^21^, ^22^, ^23^ complex intervention development guidance,^24^ kidney patient decision aids,^15^^,^^16^ guidelines on end-of-life and palliative care,^25^, ^26^, ^27^ and guidance on designing written information to ensure that the text is readable, relevant, and language used is accurate and value-free.^28^^,^^29^ To ensure rigor, accuracy, and readable content, feedback was sought from co-applicants, the independent advisory committee, and PPI teams. Wider, national stakeholder feedback was gathered from physicians specializing in kidney disease, palliative care and geriatric medicine, dialysis nurses, predialysis nurses, physiotherapists, occupational therapists, counselors, and dieticians, by providing written feedback on a draft of the guide. Graphic design and proofreading services were employed to ensure the guide was produced to a high standard.

### Research approvals

Local Research Ethics and Health Research Authority approval was granted on July 23 and August 4, 2020, respectively (IRAS project ID: 277023).

## Results

In total, 18 adults with kidney failure, 5 family members, and 4 bereaved family members were interviewed. Participants with kidney failure had a median age of 76 years (range 58-88), and 6 (33%) were female. The main characteristics of the sample are summarized in Table 1. Names used to support quotations are pseudonyms.

**Table 1:** Characteristics of the Sample

	People with Kidney Failure N (%)	Carer N (%)	Bereaved Carer N (%)
Age, y, mean (range)	76 (52-88)	59 (54-65)	77 (66-88)^a^
Sex, female, N %	6 (33)	4 (80)	3 (75)
Treatment choice, N %			
Hemodialysis	11 (61)	2 (40)	
Peritoneal dialysis	2 (11)	-	2 (50)
Conservative management	4 (22)	3 (60)	2 (50)
Undecided	1 (5)	-	

a Missing data.

### Part 1: Views and Experiences of People With Kidney Failure, Family Members, and Bereaved Family Members

Five main themes from the interviews are described.

#### How, When, Where, and With Whom End-of-Life Conversations Take Place

Most participants had not had a conversation about end-of-life treatments and care with a kidney health professional. However, they valued time and open and honest conversations with their kidney team and recognized that health professionals can find end-of-life matters difficult to discuss.

“Certainly, the impression I’ve got is that it’s the other person who finds it more difficult to talk about it than me. I got that impression; we all got that impression with Dr Wilson.” (Jim, in his 50s, hemodialysis)

Preferences varied for the circumstances within which conversations took place. Some felt that discussions should ideally be held in a private room, with members of their kidney team and family members present. Others felt that having a conversation about end-of-life when receiving hemodialysis in hospital would be acceptable. A few participants saw a role for peer-to-peer discussions with other people with kidney failure who were trained to speak on these matters.

“… which may be a role for a volunteer. … I would quite like somebody who may not be a professional, but [has] an understanding, so maybe a volunteer who would…talk it through.” (Jane, in her 70s, hemodialysis)

Preferences for the timing of this conversation also varied. Some participants expressed a wish to discuss future care and treatment early in the disease pathway, whereas others did not wish to consider these topics until there was a deterioration in their health. Some dialysis recipients felt that nursing staff were too busy or not adequately trained to have end-of-life discussions on the dialysis ward and that the opportunity to have these conversations had been missed once treatment had commenced. A few participants recognized the difficulty in knowing when the “right time” was to hold such conversations.

“Is this the right time, when I’m feeling OK and look at it logically, or is it when I start to feel sick and ill and, you know, I’m probably not in the right place to make those decisions? I think it’s a very fine line between the two.” (Mary, in her 80s, undecided)

Providing a warning and/or explanation as to why a conversation was necessary before an end-of-life discussion took place was described as important. Asking permission to have the conversation and checking the appropriateness of the location was exemplified by a carer describing the distress caused by a discussion being conducted on a hospital ward with no warning.

“So, we were quite shocked, shocked and upset that that was the topic, and even more shocked and upset that they did it with the curtain round us and no privacy.” (Louise, in her 50s, carer)

#### End-of-Life Treatment and Care Preferences

Although many participants had not had a formal discussion with their kidney team about end-of-life issues, many expressed clear preferences for the care that they would like to receive, including where they would like to die, their preference for pain medication, what happened to their body after their death, and whether or not they received resuscitation.

“I don’t want a ‘do not resuscitate’. If they can get me back to life, get me back to life. I’d much rather be alive than dead.” (Simon, in his 80s, hemodialysis)

Few people had a documented advance care plan. However, the importance of having one in place was highlighted by a bereaved husband. They described how an advance care plan assisted them in advocating on behalf of their partner to family and health professionals, and that having an advance care plan in place provided reassurance and confidence that they were carrying out their partner’s wishes in accordance with their preferences.

“I said to one of the children, ‘Well, she doesn’t want to go into hospital again.’ And son says, ‘Well, she must go to hospital, Dad.’ I said, ‘No, no, no, she doesn’t want to, and the piece of paper says she’s not supposed to.” (David, in his 70s, bereaved carer)

#### The Role of Family Members in End-of-Life Kidney Management

Some people with kidney failure had engaged in a conversation and/or made plans about end-of-life treatment and care with their spouse and/or children. Typically, these discussions included expressing a preference for location of care, funeral arrangements, financial matters, and documenting wishes in a will. Where discussions had not taken place between family members, people with kidney failure spoke of an implicit understanding that death was inevitable and/or likely to happen soon. In other instances, bereaved family members spoke about not understanding how close the person they cared for was to death and a lack of discussion about death and dying with them.

Family members described how they were involved in attending hospital appointments, liaising with palliative care and general practitioner (GP), making joint decisions about treatment, and assisting in daily care and social routines. Family members talked about their own support needs, which included emotional support, information about death and dying, respite care, and bereavement support. A few people described that they felt their needs as a carer were overlooked and that any support available would have been welcomed. In the absence of additional/written information about end-of-life issues, people sought this information themselves by searching the internet and contact with kidney charities. Family members described the importance of the GP and palliative medicine team in supporting their caring role in the last months of their family member’s life.

“Then it was so lovely, the palliative care team came round and actually, the woman, the doctor, my dad lives in Suffolk, and the consultant all met him. He was totally aware that he was going to end dialysis. And they put in so many services, it was amazing.” (Melissa, in her 60s, carer)

#### Expectations of Stopping Dialysis

A minority of people with kidney failure and family members described conversations with a kidney health professional about stopping dialysis. A few people felt that these conversations should happen earlier in a person’s illness because of the consequences of stopping and with the knowledge that people may lose capacity to make decisions. Understanding and accepting the consequences of stopping dialysis was described as difficult and required courage; some expressed disbelief. Some recognized from seeing others on treatment, that there may come a point where there was little advantage to continuing treatment.

“There’s a lot of people there who are brought in by wheelchair or who are with two sticks and obviously some of them have lost the mental capacity. I just think, ‘Why do they carry on?’ It’s not as if they’re young people.” (Jane, in her 70s, hemodialysis)

People receiving dialysis were aware that if they stopped treatment they would die, this was described in stark terms by stating, dialysis is “waiting on death row” *(Henry, in his 70s, hemodialysis)* and that stopping had “dire consequences” *(Amy, in her 50s, carer)* and “your days are numbered” *(George, in his 70s, hemodialysis).* They spoke about their experiences of seeing other people on the ward become more ill and stop attending dialysis. Some had questions about why this was. Although they understood that nurses were unable to provide detailed information, they wished to understand more about the circumstances in which people had died, and/or receive psychological support when receiving dialysis on the ward.

“…it’s difficult sometimes to get information from people, you know. You might’ve known someone, dialyzed with them, same room as ’em three times a week for, say, four or five years, and you say, ‘What happened to so and so?’ ‘Oh, confidential information.” (Jim, in his 50s, hemodialysis)

Stopping dialysis was seen as a way of being in control of one’s death. Others felt that stopping treatment was akin to dying by suicide, and some described wanting to receive dialysis as long as possible. Some family members recognized that dialysis was no longer able to sustain the health of the person that they cared for and/or that their comorbid conditions affected their ability to cope with dialysis.

“…You don’t need an assisted suicide when you’re on dialysis. You just give up dialysis, and you’re gone.” (Henry, in his 70s, hemodialysis)

#### Beliefs About Death and Dying

People held various beliefs about their own mortality. Some people were optimistic about the outlook of their illness despite being elderly and dependent on others for their care. Others predicted that another illness such as a heart attack would be more likely as a cause of death than their kidney failure.

“I know that there’s actually no reason why the kidney, the fact that my kidneys are hardly functioning at all, there’s actually no reason, with hemodialysis, why that should kill me. If I do die suddenly, it will be almost certainly because one of my other underlying conditions has suddenly provoked something.” (William, in his 70s, hemodialysis)

Some appeared comfortable discussing death, had a fatalistic approach, and viewed death as a relief. A few people had specific questions about what it was like to die from kidney failure. Some were fearful of a painful death and did not want to suffer, whereas others anticipated that death would be painless and that they would be supported by health professionals, have their pain controlled, and become more tired in the last days of life.

“I think you would want to be told, ‘It won’t be horrible. We will make sure that you’re comfortable. You’ll be in no great pain or distress’ if it’s true.” (Rose, in her 70s, conservative management)

### Part 2: Resource Content

The guide aims to build on existing skills and improve confidence around having conversations about future treatment and care (Item S1). It is for use by multi-agency health professionals, primarily renal physicians and nursing staff, but also palliative care staff and GPs. In recognition of end-of-life planning and decision making conversations not being a part of routine kidney care practice, we used the phrase “difficult conversation”: in the guide’s title, defining these as any discussion with people with kidney failure and their families around treatment and care toward the end-of-life. The phrase was chosen for consistency with a similar guides^11^^,^^12^ and based on our experience of interviewing health professionals on this topic. Since publication, we recognize there has been a move away from the use of this phrase.^30^ We recognize a need for research to identify terminology to adequately describe what we have called “difficult” conversations. Inclusive work with people living with long term conditions to choose meaningful and helpful language is needed and will be conducted during a planned update of our guide.

**Figure 1:**
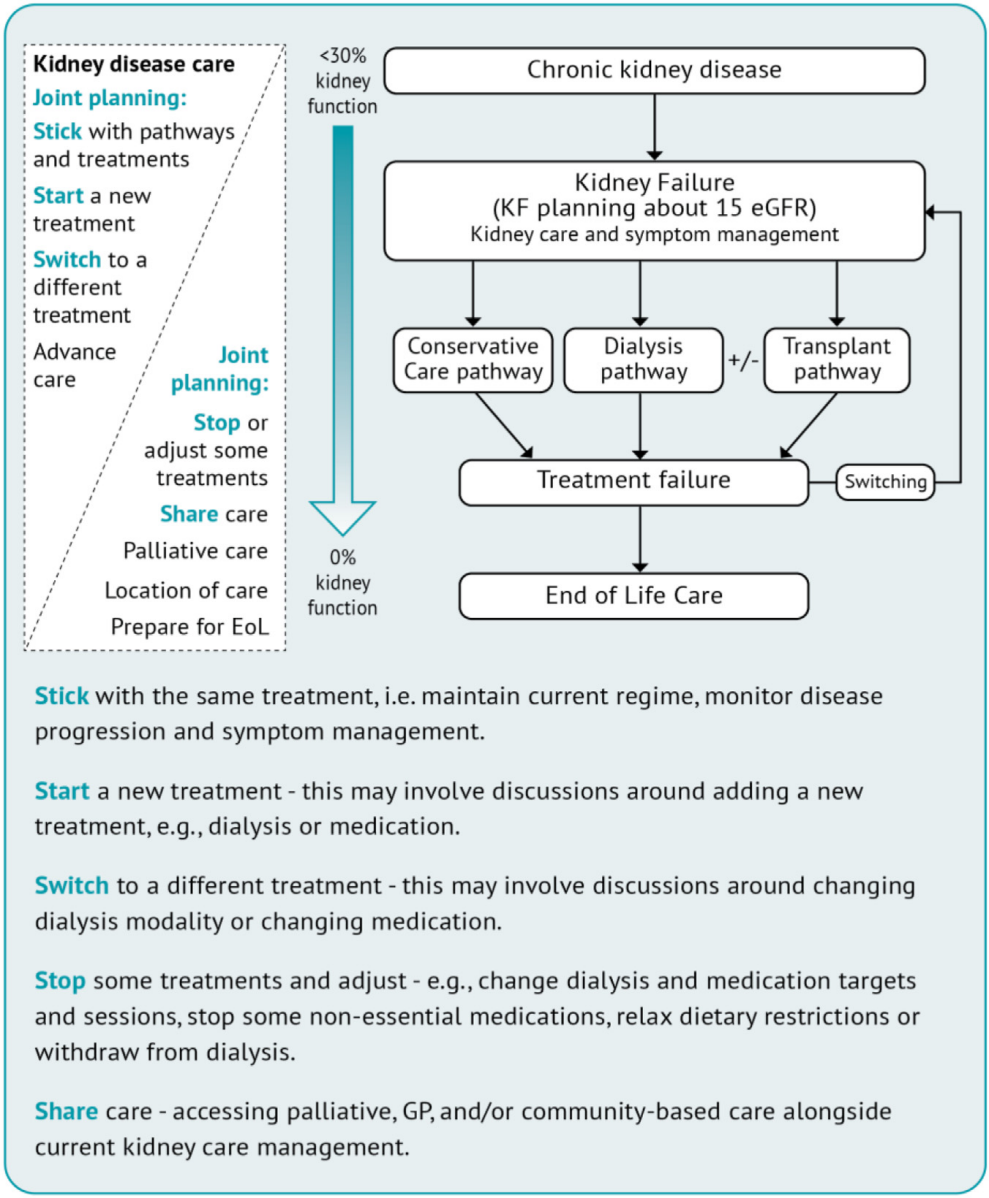
Treatment pathways decision map: showing changes in treatment and care as kidney disease progresses.

Content was developed by including (1) themes from the analysis to structure each of the 6 sections; (2) direct quotations from participants to supplement each section, (3) prompts to help share reasoning and understanding during the consultation^8^; (4) tips for holding a conversation, tables with key ideas, strategies and suggested language, and recommendations for next steps of care^21^^,^^22^; (5) a treatment pathways decision map to show changes in treatment and care as kidney disease progresses^16^; (6) the “5S’s” that were developed to describe the different treatment and care options available to people as their kidney disease worsens (Fig 1); (7) guidance on the production of quality health information^28^^,^^29^; (8) feedback sought from the project team and advisory group and PPI team; and (9) wider, national and international feedback from approximately 15 health care professionals, people with kidney failure, and academics. Feedback was wide ranging and included consideration of language used for “stopping dialysis,” use of speech bubbles to illustrate participant quotations, inclusion of bullet points, and information relevant to families/carers.

Since December 2022, the guide has been available as an open access resource at Kidney Care UK’s website (https://tinyurl.com/ss9f9dt9). Over a 6-month period, the webpage received 542 unique user visits, the guide was downloaded 163 times, and a 150 color copies were distributed at conferences and to kidney units. The guide has also been adapted for use in Denmark as part of an shared decision making intervention supporting people with kidney failure.^31^

## Discussion

This study describes in-depth and the views and experiences of people with kidney failure and their (sometimes bereaved) family members, engaging in end-of-life kidney care management conversations to plan future treatments and care. With high annual mortality, and short prognosis after dialysis cessation, we believe these discussions are important, and we developed the resource to empower professionals to engage in these difficult conversations with people with kidney failure.

Themes generally confirmed those found in prior research. In general, people with kidney failure who we interviewed were aware of the severity of their illness and the consequences of stopping dialysis^32^ and valued the opportunity to discuss end-of-life topics with health care professionals,^33^ but they had varying views around how/when these conversations should occur. This implies multiple opportunities for them to take place but also mandates their accurate recording for review within renal services and beyond.^34^

Some people we interviewed felt that early discussions about discontinuing dialysis were necessary, and opportunities to discuss end-of-life and the consequences of discontinuing treatment are lost once dialysis has commenced.^35^ Views about death and dying varied. Some expressed uncertainty about the progression of their illness and the experience of death and were fearful of pain and discomfort at the end-of-life. Others accepted their fate and felt that their comorbid conditions might be more serious than their kidney disease. Given that adults in the United Kingdom aged over 65 undergoing dialysis have an annual mortality rate of approximately 20%^35^ and that most people who discontinue dialysis die within 7-10 days,^36^ presenting verbal and written information in a balanced, unbiased way and starting conversations earlier in the illness trajectory may help people with kidney failure engage in ongoing discussions and allow better preparation for and engagement in decision making about future treatments and care.^37^

Among carers, caregiver burden was considerable, and we, like others, found impaired quality of life for those caring for people treated with dialysis and conservative management^38^ and a desire for more information and support for their caregiver role.^39^ We found that not all people with kidney failure discussed end-of-life topics with their family member who acted as their carer. Although family members are increasingly included in interventions designed to support treatment and end-of-life decision making,^40^ people with kidney failure can feel pressured to continue treatment or fear burdening family members.^41^ Further research should investigate how best to reconcile these issues and the types of interventions that might best support family members of people with kidney failure, or proxy decision makers, in the last months of life and post bereavement.

**Figure 2:**
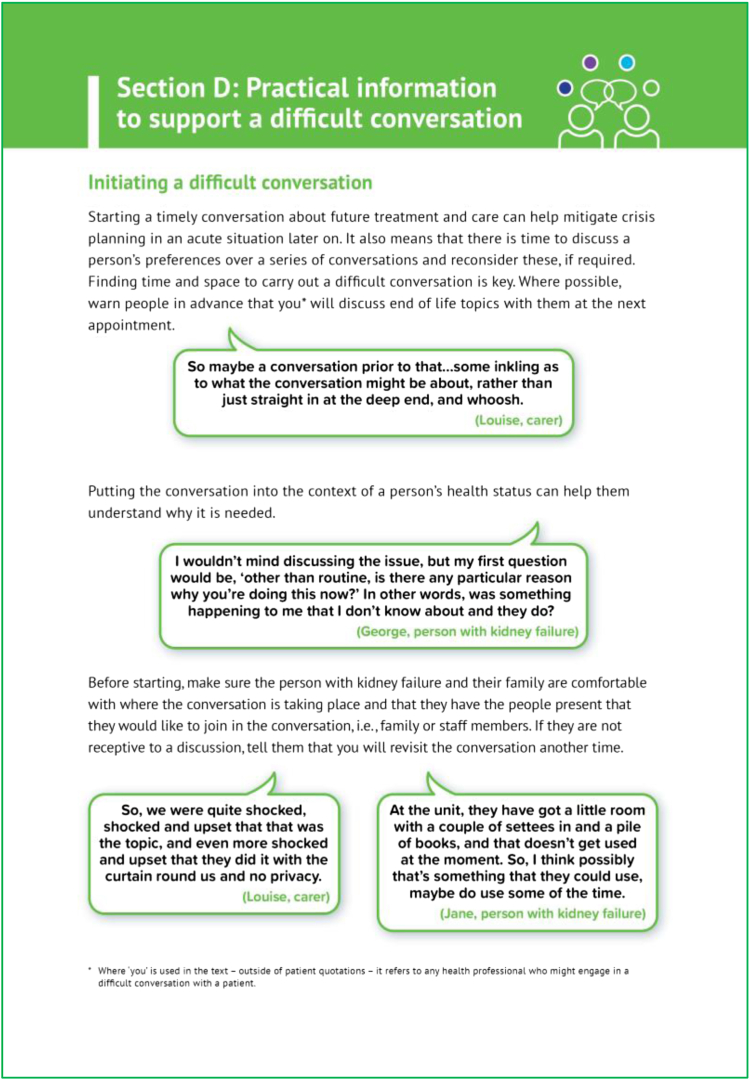
Extract from the “Difficult Conversations” guide showing examples of quotations from patient and family members.

The use of advance care planning interventions and The Serious Illness Conversations Guide within UK renal units are in their relative infancy.^10^^,^^42^ The Serious Illness Conversations Guide informed the content of our resource; however, to our knowledge, ours is the first bespoke, kidney-specific guide designed available and informed by the lived experience of people with kidney failure and their carers. A user-centered design was employed with independent patient and carer input from study inception, themes from the qualitative interviews informing the guide’s structure, and the inclusion of explicit patient and carer quotations incorporated throughout the resource (Fig 2). Resources used to support kidney care management and decision making are seldom developed using such rigorous methods.^37^ Although patient narratives can potentially bias decision making,^43^ this resource has a different purpose in the shared decision making process. The patient/carer narrative provides authenticity and relatability and makes explicit new insights, thereby empowering kidney health professionals to initiate and engage in end-of-life conversations.

Study recruitment was challenging. The COVID-19 pandemic mandated recruitment by letter and many declined to take part. In the future, incorporating a screening question on reasons for non-participation may provide useful insight. Participants were mainly White, British, and receiving hemodialysis. Interpretation of the data must take into consideration these limitations and reflects pervasive difficulties identified by others in recruiting diverse populations into research studies.^44^ Participants took part in interviews between May 2021 and March 2022 after the COVID-19 pandemic. Questions posed were broad and not time-specific. In addition, we did not ask about the impact of COVID-19. Few participants mentioned the pandemic; however, it may have implicitly affected participants perceptions of planning for future treatment and care.

Kidney teams have expressed an interest in how to implement the guide in practice and within their broader communications training packages. To address, this we are developing an interactive online and in-person training package to accompany the resource and we have an agreement in principle from the Association of Nephrology Nurses (ANN-UK) to incorporate online training into their new online kidney care course. Future work will explore how useful the guide is by United Kingdom-based clinical teams and how it affects service delivery.

## Supplementary Materials

Supplementary File (PDF) Item S1.
