# Supplementary material for: Development of a Resource for Health Professionals to Raise Advance Care Planning Topics During Kidney Care Consultations: A Multiple User-Centered Design
Source: Kidney Med. 2024 Jul 18;6(9):100874. doi: 10.1016/j.xkme.2024.100874 (PMC11359737; doi:10.1016/j.xkme.2024.100874)
Supplement: Supplementary File (PDF) — Item S1. [file mmc1.pdf]

Difficult Conversations:

Talking with people about kidney failure, end of life and advance care planning

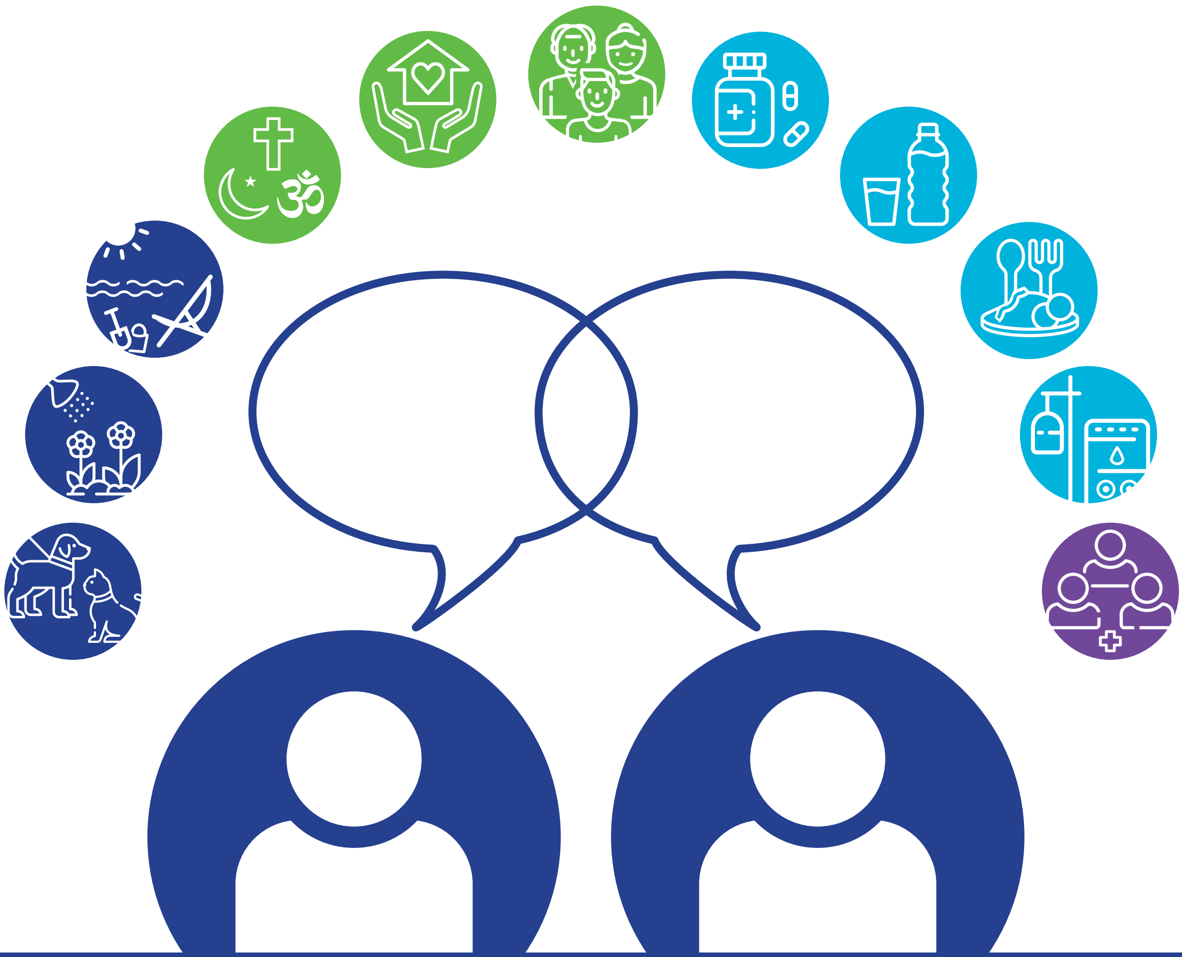

A guide for health professionals

About the Booklet

This booklet is a guide for health professionals engaging in 'difficult conversations' with people with kidney failure and their families about the treatment and care that they would like to receive towards the end of life. For the purposes of this booklet, a 'difficult conversation' refers to any discussion of these issues involving health professionals, people with kidney failure, and their families.

Engaging in difficult conversations about a person's preferences for future treatment and care can help improve patients' and families' experience of end of life kidney care, and the standard of care provided by healthcare services (Box 1). The booklet aims to build on health professionals' existing skills to help make difficult conversations a more ordinary and frequent part of practice.

Box 1: Reasons to engage in difficult conversations as part of kidney care management

Benefits for people with kidney failure and their families:

- improves knowledge about what treatment and care will be available in the future,
- reduces anxiety about what to expect from their end of life health and care,
- allows for more realistic expectations about possible outcomes of treatments,
- increases the likelihood of receiving palliative care and effective symptom control.

Benefits for kidney health professionals:

- supports health professionals to challenge their current practice and assumptions about what they feel is best for their patients,
- improves understanding of people's needs and preferences in the context of their lives,
- facilitates patient-clinician decision making around goals of care and end of life options,
- improves communication among all parties by creating a written record of a person's goals and expectations,
- increases skills, confidence, and morale.

Benefits for kidney services:

- improves allocation and co-ordination of all relevant services,
- sharing information between parties and producing a plan in advance helps to mitigate 'crisis' planning in acute situations.

Booklet development

This booklet is based on the lived experience of people with kidney failure, their family members, and bereaved family members who cared for someone with kidney failure.

We interviewed people to understand their views on important issues when approaching the end of life that impact on their experience, decision making, treatment and care, and people's views and experience of caring for and supporting someone towards the end of life.

In total we spoke to 27 people – 18 people with kidney failure receiving haemodialysis, peritoneal dialysis, and conservative care, five family members, and four bereaved family members. Of these, 13 were women and 14 were men aged between 52 and 88 years old, with an average age of 72 years. People were interviewed in their own homes, in hospital, via a video link, and on the telephone, and were based throughout the United Kingdom.

The interviews were recorded and analysed to identify the main themes that were discussed. These themes helped develop the content and structure of the booklet. Although this booklet cannot cover everyone's views, a selection of experiences are quoted. Pseudonyms are used throughout the booklet for anonymity.

A multidisciplinary team of researchers, health professionals, and patient partners with expertise in kidney medicine and palliative care helped to develop this booklet. More details about the study on which it is based can be found on page 34.

Booklet endorsed by:

Association of Nephrology Nurses UK

Disclaimer:
Every effort has been made to provide accurate information at the time of publication (2022).

Date of next content review and update: January 2025.

Contents

This booklet can be used for reference and guidance before, during, and after patient consultations [1,2,3,4]. The booklet is for any health professional caring for someone with kidney failure and their family, including kidney doctors and nurses, geriatricians, palliative care doctors and nurses, social workers, allied health professionals, healthcare assistants, care home staff, general practitioners, district nurses, and community matrons.

To help focus on particular aspects of difficult conversations, the booklet is split into the following sections:

Section A: Difficult conversations are valued but often don't happen 5

Section B: Difficult conversation scenarios 6

Section C: Difficult conversation topics 10

Section D: Practical information to support a difficult conversation 15

Section E: Supporting services and information 26

Section F: Further information 29

The booklet contains:

Information about holding difficult conversations – some sentences have numbers in brackets, e.g., [1,2], linking information to references or sources for facts, which are listed on pages 32 – 33,

Quotations from people with kidney failure, family members, and bereaved family members about their experiences of kidney failure and what is important to them about future care and treatment,

Tables* with key ideas, strategies, and suggested language for holding difficult conversations and suggested language to make recommendations for next steps in care,

A treatment pathways decision map** showing different care pathways over time for people with kidney failure.

* Adapted from Ziegler L et al., STEP resource for health professionals talking to people in oncology about prognosis and end of life. University of Leeds, 2020.

** Adapted from Winterbottom A, Mooney A, Russon L, Hipkiss V, Williams R, Ziegler L, Finderup, J, Bekker HL. The Yorkshire Dialysis decision and conservative care aid booklet (YoDCA), 2020. (Accessed via kidneyresearchyorkshire.org.uk)

Section A: Difficult conversations are valued but often don't happen

People with kidney failure experience a high symptom and treatment burden, multiple long-term conditions, and associated poor prognosis [5,6,7]. In the United Kingdom, adults aged over 65 undergoing dialysis have an annual mortality rate of approximately 20% [8]. Despite this, advance care planning, hospices, and palliative care are underused by kidney services. Compared with other chronic illness populations, people with kidney failure experience high rates of hospitalisation, intensive care unit admissions, and other intensive treatments in the last months of life [9,10].

Some people with kidney failure experience a prolonged decline in their health; other people have a short deterioration or sudden death [11]. The unpredictable disease trajectory, potential for overtreatment, and poor access to supportive palliative and end of life services highlight the importance of sensitive and timely conversations between health professionals and people with kidney failure. Such conversations can help to ensure that, regardless of age and treatment modality, people's preferences for treatment and care towards the end of life can be met [5,11,12,13,14].

These difficult conversations are not taking place as part of routine kidney care practice. Most people we interviewed had not discussed end of life treatment and care with a health professional. This may be because health professionals fear upsetting their patients and/or lack confidence, skills, and training in this area [4,15,16]. People with kidney failure who we interviewed recognised these issues, and told us that they valued time, honesty, and open communication when discussing these matters.

Certainly, the impression I've got is that it's the other person who finds it more difficult to talk about it than me. I got that impression; we all got that impression with Dr Wilson.

(Jim, person with kidney failure)

And as we know, in the NHS, this subject is, we're trying to open up to people, so they talk about death and stuff like that, yeah? It's so taboo.

(Hussain, person with kidney failure)

We had a very close and understanding relationship with Dr Jones. He always respected her wishes, and we understood each other extraordinarily well. He was the only one who was holding it together and he kept in touch with us. Dr Jones was good.

(David, bereaved carer)

And it's nice to be informed, you know. The doctors take the time to explain to you. I know they're busy and that, but it does help the patient.

(Victor, person with kidney failure)

Section B: Difficult conversation scenarios

This section describes two approaches to engaging people with kidney failure in a difficult conversation. Kidney health professionals often develop close relationships with their patients over month or years, which should enable more honest conversations. However, it can be hard to know when it is the 'right time' to discuss future treatment and care. People with kidney failure vary in their preferences for how, when, and with whom these conversations should take place.

Is this the right time, when I'm feeling OK and look at it logically, or is it when I start to feel sick and ill [and] I'm probably not in the right place to make those decisions?

(Mary, person with kidney failure)

1. Advance care planning

Talking to people about their goals of care and expectations for the future can be a step towards producing an advance care plan. Advance care plans can be made at any time and offer people the opportunity to make a written plan, accessible by any health professional, about their goals, values, and preferences for future treatment and care if they become unable to express their wishes when their health worsens. People can change their minds about their decisions if their views and circumstances change. Advance care plans do not replace the clinical decision making that takes place when a decision is required. A treatment would not be withheld if it was felt it would be beneficial to someone.

Not everyone will want to make an advance care plan, but they can have repeated chances to make one at different stages of the care pathway, whenever goals of care and future treatment are discussed. Although few people we spoke to had formally made an advance care plan, many people expressed clear preferences for care and had made their own plans, often in conjunction with their spouses. Advance care plans can help kidney services to know people have spoken with family members about the end of their life, and what they decided.

I said to one of the children, 'Well, she doesn't want to go into hospital again.' And son says, 'Well, she must go to hospital, Dad.' I said, 'No, no, no, she doesn't want to, and the piece of paper says she's not supposed to.'

(David, bereaved carer)

We'd already made our wills. We'd already planned our funerals. We've put everything in place for our three children, and that was something, you know, I was pleased I'd got out of the way.

(Mary, person with kidney failure)

2. Transitions in kidney care management

Opportunities to hold a difficult conversation can include when the management of a person's kidney failure might be changing due to a decline in their health status and/or when their kidney failure impacts on their ability to do everyday activities. People we spoke to recognised that changes in their health and treatment mark suitable times to discuss preferences for future care and treatment.

I think to do it right at the beginning. I think they should put it out there in the open because of what the condition is.
(Melissa, carer)

I don't want people to be frightened of saying, 'I'm sorry, George, you've got a couple of weeks left.' I would rather them tell me that, because, obviously that's going to affect my family.
(George, person with kidney failure)

We would like medical staff to have a conversation with the family on admission to hospital about his end of life wishes. We feel that they probably knew he was at the end of his life then.
(Pearl, bereaved carer)

Involving family members in difficult conversations

Family members of people with kidney failure may be actively involved in their daily care routines, including attending hospital appointments, and sharing treatment and care decisions. People with kidney failure may want family members to be included in conversations about future care and treatment. They may not want to have a difficult conversation if key family members or health professionals are not in the room (Table 1).

Yeah, I think really this conversation would start with, obviously, the doctors and nurses and with my family. And they're the only other people that need to be involved in the conversation, or palliative nurses.
(Hussain, person with kidney failure)

These conversations should be had with the family members present because, let's face it, we have to remember that the people who are most affected, I mean apart from the patient himself, are his closest relatives, his spouse or parents or children or whatever.
(William, person with kidney failure)

People with kidney failure also have conversations with their spouses and families about their end of life care preferences and rely on them to communicate on their behalf when necessary. This may mean that a difficult conversation involves talking with a family member, not the person with kidney failure.

I had to go away and just think to myself about what did I think...Was I switching her off? And I realised that it was, in fact, it was her right to refuse medication. She didn't have to have it...And so I told Angela, and she knew, so that's what we did.

(David, bereaved carer)

She talked about that, she hasn't written a will, but her family know what she wants.

(Joan, person with kidney failure)

Table 1: Suggested language for making a recommendation about involving others

Suggested language
"Since your family doesn't really understand yet how ill you are, I recommend that you bring them in and we talk with them together about where you are and what is likely to be ahead." *"Would you like to bring along your [family member/carer] and/or I can organise [health professional] to be present next time and we can discuss these issues then?"*

Treatment pathways decision map

The treatment pathways decision map on page 9 is an overview of the different types of treatment options and care plans that can happen as people's kidney failure gets worse. It helps people make plans with health professionals before they become ill. The transition points shown by arrows often coincide with the need to discuss current and future care options. For example, people can be signposted to these topics when they are making choices about renal replacement therapies and conservative management.

This visual aid can be shared with a patient during a consultation. The map can be used to explain how a person's treatment fits within the context of their illness, how their care will change over time, and when they might consider sticking, starting, switching, stopping, or sharing treatment and care. See 'reviewing treatments and care' on page 12 for a more detailed discussion of these options.

This conversation can be supplemented by handing out the YoDCA booklet to people, to take home and share with family members - see page 30.

Treatment pathways decision map: Changes in treatment and care as kidney disease progresses [23, 24]

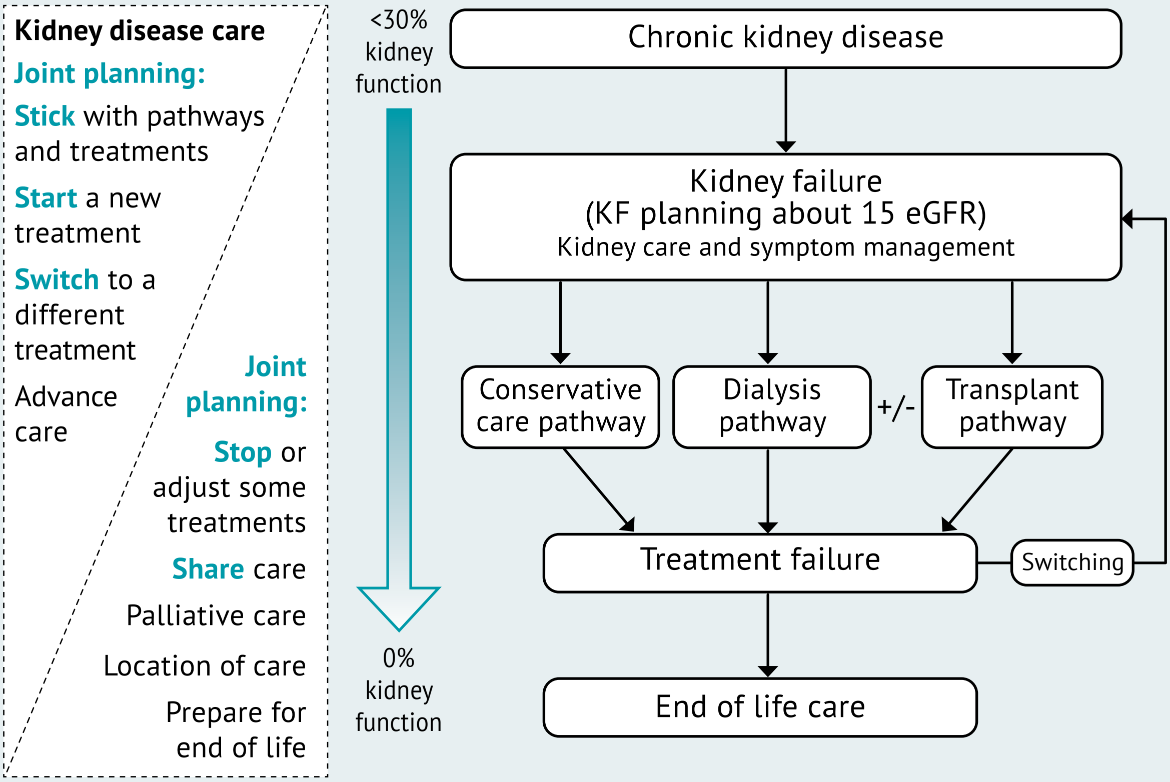

Stick with the same treatment, i.e., maintain current regime, monitor disease progression and symptom management.

Start a new treatment – this may involve discussions around adding a new treatment, e.g., dialysis or medication.

Switch to a different treatment – this may involve discussions around changing dialysis modality or changing medication.

Stop some treatments and adjust – e.g., change dialysis and medication targets and sessions, stop some non-essential medications, relax dietary restrictions, or withdraw from dialysis.

Share care – accessing palliative, GP, and/or community-based care alongside current kidney care management.

Section C: Difficult conversation topics

As a person's health may decline over a protracted period, there can be many times before the last few days and weeks of life when conversations about future care and treatment can happen. Discussions may centre around what is important to a person to maintain their quality of life, reviewing existing treatments, end of life care options, and decisions that will affect family and friends after the person has died. This section focuses on each of these topics in turn.

Maintaining quality of life

Discussing what is important to a person in their everyday life and the likely outcome of treatment helps to set goals, Table 2 (page 11) and have realistic expectations about future care [17,18], Table 3, (page 11). Some topics that were important to people we interviewed included:

- understanding what constitutes a good quality of life for a person with kidney failure,
- how kidney disease is likely to progress on the current kidney management plan,
- establishing that dialysis is a treatment for kidney disease and not a cure for the illness,
- understanding that all treatment may become less effective over time,
- understanding the consequences of stopping dialysis.

I think they need to have that conversation about what dialysis is. It's keeping you alive, and a few other things, and I think it's not really talked about.
(Melissa, carer)

Because I know somebody else who was on dialysis, he just stopped coming in, and he stopped his treatment, and I heard that a week later he'd died.
(Victor, person with kidney failure)

'Do you want to be resuscitated? I wouldn't necessarily recommend it.' And I thought, 'You cheeky monkey! Quality of life is how I look at things, not how you look at them.'
(George, person with kidney failure)

They've shied away from saying, 'Ultimately, these treatments don't last forever' because they want people to be hopeful and optimistic.
(Rose, person with kidney failure)

I appreciated his frankness. The nephrologist said quite frankly that if I didn't go on to dialysis, my life expectancy was six months.
(William, person with kidney failure)

Table 2: Strategies and suggested language for goal setting

Goal setting	
"How do you want to spend the last two or three years of life or whatever? Like you say, it's quality, not quantity." (Amy, carer)	
**Key ideas**	**Strategies and suggested language**
Set goals – this can help identify circumstances in which sticking, starting, switching, stopping, or sharing treatments and care might be appropriate.	When possible, discuss early on a person's hopes, goals, and future health states which might impact on their desire or ability to carry out treatment.<br>*"Can you tell me about things that you do in life that you feel are important and would like to continue doing?"*<br>*"I know that you like to go bowling once a week. If in the future you are no longer able to do that, I wonder if we should think about adjusting your treatments."*

Table 3: Strategies and suggested language for dealing with unrealistic expectations

Unrealistic expectations	
"But as far as she's concerned, she isn't going to go downhill rapidly. She's going to hit her hundredth birthday." (Violet, carer)	
**Key ideas**	**Strategies and suggested language**
Being a "fighter" and being "fine" have many meanings.<br>A person may believe that treatment can go on for many years more than is realistically possible.	People may need permission to think differently about coping with their illness.<br>Explore what a person means by re-framing:<br>*"Tell more more about how you are not letting the disease 'get the best of you.'"*

Reviewing treatments and care

As a person's disease progresses, to continue meeting their goals and preferences, it may be necessary to review the current treatment regime. It may be helpful to give a person the opportunity to confirm the circumstances in which they would want to consider making decisions about future treatment (Table 4). People with kidney failure may not be aware of the treatment and care options available, which include:

- **stick** with the same treatment – i.e., maintain current regime, monitor disease progression and symptom management.
- **start** a new treatment – this may involve discussions around adding a new treatment, e.g., dialysis or medication,
- **switch** to a different treatment – this may involve discussions about changing dialysis modality, or changing medication,
- **stop** some treatments and adjust – e.g., change dialysis and medication targets and sessions, stop some non-essential medications, relax dietary restrictions or withdraw from dialysis.
- **share** care – access palliative, GP, and/or community-based care alongside current kidney care management – see page 26.

Table 4: Suggested language for discussing changes to treatment or care

**Suggested language**	
**Stick** with treatment	*"Based on what you've said about how you can still fit [activity] in, I recommend that we keep your current treatment regime as it is for now, while you are still able to keep doing [activity]."*
**Start** a new treatment	*"So that we can try and manage some of the symptoms of your kidney disease, I wonder if we should discuss with other members of the team and your family about starting a new treatment."*
**Switch** to a different treatment	*"It sounds like carrying out [treatment] is impacting on your ability to do [activity]. I suggest that we discuss with other members of the team and your family whether you can try a different treatment so you can keep doing the things you enjoy."*
**Stop** some treatments	*"There might come a time in the future when you think about whether you want to stop doing dialysis as often. I wonder if it would be helpful to take time now to consider what it would mean if you reduced the number of times a week that you have this treatment."*
**Share** care	*"Based on what you have told me about how much you are struggling with [issue], I suggest that we discuss with other members of the team and your family whether it is appropriate to share care with other services that can support you. This may include providing care in your home or via a hospice."*

End of life care options

People we spoke to had clear preferences for the type of care they wished to receive towards the end of life. They stated that they weren't afraid of death and accepted it as inevitable. People may hold a wide range of cultural, religious, and ethical views that impact on their preferences for end of life care, Table 5 (page 14). Topics that were important to people we interviewed included:

- whether they were nearing the end of their life and how long they may have left,
- what it is like to die from kidney failure,
- location of treatment, care, and death – i.e., home, hospital, or hospice,
- decisions about treatment and care they do or do not want – e.g., resuscitation and sedation,
- faith, cultural, or spiritual beliefs that may influence care and death preferences.

I'm not afraid of death...My father used to say to me, 'Live every day as if it's your last because one day it will be.' I live it that way...I'm a fatalist. It's going to happen to me one day, as it is to everybody else.
(Simon, person with kidney failure)

I've got this marvellous family that keeps me going. I'm looked after so well. So basically, I don't see myself as being end of life because of that.
(Henry, person with kidney failure)

I did read something somewhere, and it suggested that you might only live for a very short time, two or three weeks. It won't be, whoops, tomorrow you go. You'll get poorly for a while, and they will support you through that.
(Jane, person with kidney failure)

The thing is that I don't want to die in hospital. I don't know why I've got such a prejudice against it. Lots of people do, don't they, you know? But I just don't want to do that.
(Jim, person with kidney failure)

I don't want to be sedated...I need to have a choice right to the end for my treatment for my pain or anything.
(Hussain, person with kidney failure)

When he asked Dr Ward in the very beginning, 'How will I know when I'm getting worse?', and she said, 'You will just get more and more tired. It's not painful.' And that's really what happened.
(Pearl, bereaved carer)

Table 5: Key ideas and suggested language for discussing religion, culture, spirituality, and existential issues

**Addressing religion and culture**	
"So the main thing in our [culture] is that when a person dies, they prefer to go as a whole and not have a post-mortem, to try to get my body [and] bury me as quickly as possible rather than putting me in the freezer." (Hussain, person with kidney failure)	
**Key ideas**	**Suggested language**
Faith plays a big part in how some people think about end of life decisions.<br><br>Some people won't talk about their faith unless directly questioned.<br><br>Showing understanding and respect for faith increases trust in the health professional and care process.<br><br>A person may benefit from an in-depth discussion about how their religious and spiritual beliefs might impact on treatment and care.	*"Are there key beliefs that you would want to record in your notes in terms of the care we provide?"*<br><br>*"What role does your faith play in your thinking about the care you want if you become unwell?"*<br><br>*"I wonder if you would like to discuss your beliefs and how they might impact on your future treatment and care with someone in our chaplaincy service."*

Decisions affecting family and friends after someone has died

Many people we interviewed had put plans in place for arrangements after their death to help ease the burden on their family members at this time. These included:

- naming a person to make financial, personal, and care decisions (lasting power of attorney),
- making a will, funeral plans, and arrangements for pets and family,
- decisions about donation of organs, tissue, and/or body.

My documentation says [the hospital] will take my body if it's good enough because it might be useful to them. I'm a big fan actually of direct cremations, but my husband won't hear of it.

(Rose, person with kidney failure)

I've written my will. I've stated who I want my goods to go to, and I've stated that I would like a cremation.

(Jack, person with kidney failure)

Section D: Practical information to support a difficult conversation

Initiating a difficult conversation

Starting a timely conversation about future treatment and care can help mitigate crisis planning in an acute situation later on. It also means that there is time to discuss a person's preferences over a series of conversations and reconsider these, if required. Finding time and space to carry out a difficult conversation is key. Where possible, warn people in advance that you* will discuss end of life topics with them at the next appointment.

> **So maybe a conversation prior to that...some inkling as to what the conversation might be about, rather than just straight in at the deep end, and whoosh.**
> (Louise, carer)

Putting the conversation into the context of a person's health status can help them understand why it is needed.

> **I wouldn't mind discussing the issue, but my first question would be, 'Other than routine, is there any particular reason why you're doing this now?' In other words, was something happening to me that I don't know about and they do?**
> (George, person with kidney failure)

Before starting, make sure the person with kidney failure and their family are comfortable with where the conversation is taking place and that they have the people present that they would like to join in the conversation, i.e., family or staff members. If they are not receptive to a discussion, tell them that you will revisit the conversation another time.

> **So, we were quite shocked, shocked and upset that that was the topic, and even more shocked and upset that they did it with the curtain round us and no privacy.**
> (Louise, carer)

> **At the unit, they have got a little room with a couple of settees in and a pile of books, and that doesn't get used at the moment. So, I think possibly that's something that they could use, maybe do use some of the time.**
> (Jane, person with kidney failure)

* Where 'you' is used in the text – outside of patient quotations – it refers to any health professional who might engage in a difficult conversation with a patient.

Invite people to write down any questions that they may have before, during, and after the conversation. Provide contact details so that people can get in touch if they have questions, e.g., a telephone number, postal address, or email.

> **I get home and I think, 'Oh, God, why didn't I ask that?' And then I get my notebook out, I get my pen, and I think, 'Right, what was it I wanted to say?'**
>
> **(Violet, carer)**

Focus on future care and treatment topics only, emphasising that the person controls these decisions. This will keep the conversation on track and will not overwhelm someone with extra information about other health matters. Where appropriate, at the end of the conversation, recommend next steps for care based on what is important to them. Some people may respond to these conversations by being confrontational; others may wish to avoid discussing the topic. Table 6 (page 17), Table 7 (page 18), and Table 8 (page 20) provide information on how to initiate a conversation and keep it focused.

Patient resources have been developed to help people with kidney failure engage with these complex decisions and start conversations with their family members' e.g., the Dialysis Decision Aid (YoDDA) and the YoDCA. See page 29 for links to these publicly available resources [19,20].

The use of language

Specific words and phrases used in everyday practice can unintentionally impact on the way in which information is interpreted, and decisions that people make about treatments. Three examples are described:

'Stopping treatment'. It may be appropriate to adjust dialysis targets and conduct fewer sessions each week or even stop dialysis altogether. Using phrases such as 'stopping' or 'withdrawing' from dialysis may give the impression that there will be no further treatment of a person's kidney failure. Re-framing the conversation to discuss the continuation and/or addition of medication, palliative and supportive services can help people to understand how health professionals will continue to provide support and care.

Terms used to describe illness and death. Emotive or vague terms such as 'fighter', 'brave' and 'passed away' have different meanings for different people. Avoiding metaphors and using simple, factual language can help to improve understanding, e.g., saying 'death' not 'passed away'. Rather than defining a person by their illness or treatment, i.e., 'dialysis patient' or 'kidney patient', consider emphasising the individual by describing a person as 'living with kidney disease'.

Presenting information in a balanced way. When health professionals provide information about treatments they can 'leak' their preferences for one option over another with the language that is used. For example, conservative care is sometimes described as a passive, 'no treatment' option. The use of resources designed to present information in a balanced way can help overcome this bias – see page 29.

Table 6: Key ideas and suggested language for initiating a difficult conversation

**Initiating a conversation**	
"I think I can discuss it any time. I'm pretty open-minded. I've been used to a very mixed life." (Jack, person with kidney failure)	
**Key ideas**	**Suggested language**
Describe the purpose of the conversation.	*"I know we usually talk about your blood results, but next time I want us to talk about what kind of treatment and care you might want in the future."*<br><br>*"At our last appointment, I mentioned to you that we would have a discussion today and do some planning and thinking in advance about what kind of care you might want in the future."*
Explain how the conversation fits into their health status and care pathway.	*"We know that people's kidney disease can get worse, and it is important for people to talk about these issues when they are well, so we can plan care together, and with family members."*
State benefits and support available.	*"Talking about it now allows all of us time and space to talk and think these issues through, and to include your family in our discussion, now or later."*
The 'hook' – allowing the person to remain in control.	*"I want to begin these conversations so that we are both better prepared for changes that can happen. The key is for you to think about what is important to you, and share with us the types of decisions you want to make."*
Give the person control.	*"Is this OK? If it isn't, we don't have to discuss it today. When would you prefer we discuss it?"*

Table 7: Strategies and suggested language for dealing with avoidance

**Avoidance**	
"My reaction was, 'Hello?! Just hang on a minute! I'm fine now. I don't want to talk about this!'" (Mary, person with kidney failure)	
**Key ideas**	**Strategies and suggested language**
Exploring why someone does not feel able to talk about these issues can provide valuable information that helps you provide good clinical care.	Explore their reasons for not wanting to discuss this.<br>*"Help me understand why you would prefer not to talk about this."*<br>Elicit information on how they think about planning for the future.<br>*"I'd like to understand what kind of thinking and planning you would find helpful as we think about what is ahead with your illness."*
Many people are uncertain about receiving information and discussing the future. They may want to talk about these issues but be scared of what they will hear.	If they are ambivalent, acknowledge or name the ambivalence and how difficult the situation is.<br>*"I hear you saying you know it is important to do some planning and also that you worry this process will be too overwhelming."*
People may have intense fears about the future and about dying that are overwhelming – if this is the case, finding a way to gradually introduce the subject may help the person be better prepared for reality.	*"I hear that you are feeling very scared about what is ahead, including about dying. This is something we can talk about, if you would like to."*<br>OR<br>*"I would like to refer you to see one of my palliative care colleagues – they are experts in helping people cope with illness and decisions about end of life care."*
This may be a bad time because of other difficult events (e.g., symptoms, other life stressors).	*"I understand that you have [situation] going on in your life at the moment. We can come back to this conversation at a later date."*

If a person has an anxiety disorder, it can make it difficult for them to cope with the anxiety of a discussion.	*"I think it might be helpful for you to have some extra support in dealing with your illness. I'd like you to meet with a psychologist colleague who is an expert in supporting people in coping with illness."*
The conversation may stray from the topic if the person is anxious or has other high priority issues to discuss.	Acknowledge that this is a difficult conversation and gently bring them back to the topic.<br>*"I know this is hard to talk about, but I'd like to see if we can clarify a couple of things about your worries for the future."*
If a person strays from the topic, they will usually recognise that you have an agenda and need to fulfil it within a limited time frame, if reminded.	Remind the person about time constraints.<br>*"I wish we had more time to talk about your new dog, but I would like to get back to thinking about some future planning that I think we need to do."*

Table 8: Strategies and suggested language for dealing with direct confrontation

**Direct confrontation**	
"I think it's a bit of a shock. Something like this would probably freak them out, 'How would you like to die?' 'You mean I'm dying already? I've just started on dialysis.'" (Hussain, person with kidney failure)	
**Key ideas**	**Strategies and suggested language**
People assume the health professional has bad news about their health status if they start a conversation about future care.	Provide perspective.<br>*"I can tell that this is a hard conversation to be having. Right now, you are doing well. I am bringing this up now because we do not know if and when your kidney disease will start getting worse. These conversations help us work together to prepare a plan, in case something unexpected happens."*
Conversations about the future inevitably bring up fears about dying.	Ask the person what makes them concerned.<br>*"It sounds like you are pretty worried that you might be close to the end. Please tell me more."*<br><br>Name and explore what is hard.<br>*"I know that this can be scary to talk about. What are your biggest fears?"*
The health professional's key task is to manage anxiety, emphasising what is still possible while exploring the person's concerns and the medical realities.	Be honest and as hopeful as you can realistically be.<br>*"I do think you are in a very difficult place, and your disease is worsening. I think time may be getting short. At the same time, you are still here and still very much yourself, and I would like to focus on helping you feel as well as possible so you can spend time on what matters most to you."*

Tips for holding a difficult conversation

At the start:

- schedule consultations to allow time to start a meaningful conversation,
- check that the person is happy with the location and participants,
- consider having the conversations over several consultations if time is short or if the person is easily overwhelmed.

During the conversation:

- focus on the person's quality of life, fears, and concerns,
- acknowledge and explore emotions,
- emphasise that there are choices and options (stick, start, switch, stop, and share care),
- check understanding of any medical terms and provide explanations where necessary,
- give a direct, honest prognosis if requested,
- talk for less than half the time,
- allow silence,
- avoid using the computer while talking, and make frequent eye contact,
- offer patient information leaflets and/or signpost to other information sources,
- if appropriate, propose making a referral to palliative care, allied health professionals, hospice services, advance care plan, team treatment review meeting, and/or involving the GP team,
- gather referral information from the person and explain why you need it,
- check if the person has had conversations with family members, and what plans have already been made.

Towards the end:

- allow time for questions and comments from the person and any family member/carer,
- summarise the conversation and ask the person to repeat back the summary to check that they understand,
- clarify next steps for you and for them (write these down for the person to take away if you think it will help them),
- document the conversation in the person's medical notes, using their own words to convey their perspective,
- ensure that there is another appointment booked with the same or a different service or an alternative way for the person to get in touch if they have questions.

Managing emotional responses to difficult conversations

Difficult conversations can evoke strong emotions for people with kidney failure, their family members, and health professionals. Anxiety is usually the most common feeling on both sides. Anxiety may impact on the conversation by influencing a person's words, facial expressions, body language, use of psychological defences, and affective expressions (e.g., tears, flushing in the face). Anxiety expressed by a patient can provide the health professional with a useful indicator of their state of mind. People with kidney failure and their families may also express emotions such as anger, sadness, and fear about discussing end of life topics. Table 9 (page 22), Table 10 (page 23) and Table 11 (page 24) include information on how to address these emotions during a conversation. Engage team members who have a close relationship with the person to help support both you and the person during and after the conversation.

Table 9: Strategies and suggested language for dealing with anger

**Anger**	
"Nobody really sat down with him and said, 'You must be really disappointed.' And for me as well, because I felt really, really upset." (Melissa, carer)	
**Key ideas**	**Strategies and suggested language**
Stay calm.	'I wish' responses are helpful.<br>*"I wish you were able to continue doing the things that you enjoy."*
Anger in this setting is usually about accepting the message and not directed at the health professional personally.	Explore and acknowledge the anger using neutral language.<br>*"I can see this is really frustrating. Tell me more about the frustrations you've been experiencing."*
Giving the person an opportunity to talk about their anger, and responding non-defensively, tends to be therapeutic.	Encourage the person to say what is on their mind.<br>*"As hard as it is, I want to learn as much as I can about what this is like for you, including about your frustrations."*<br>Respond non-defensively.<br>*"I can understand that you feel that I let you down. I will still work hard to do my best for you."*

Table 10: Strategies and suggested language for dealing with anxiety

Anxiety	
"I know when the doctor rang out of the blue, I said to him, 'I'm all right. I'm hoping to live a bit longer.' And, well, he probably thought I was very rude, but it was just a frightened reaction really." (Mary, person with kidney failure)	
**Key ideas**	**Strategies and suggested language**
A health professional's anxiety will increase a patient's anxiety.	Pause and settle yourself before beginning the conversation; speak clearly and at a measured pace.
Strong emotions tend to impair clear thinking.	Recognising and acknowledging a person's emotions, and allowing them time to process these, can help them to process the information and think about making a plan.
People may have other important concerns which impact on the conversation.	Being in a crisis situation, whether medical or affecting another area of life, diminishes a person's capacity to deal with anxiety. If possible, delay a difficult conversation until any crisis has passed. If this is not possible, recognise that you will have to proceed especially gently.
Express empathy and affirm a connection with the person to help reduce their anxiety.	*"I can see how hard this is for you to talk about."*<br>*"I wish we were not in this situation."*<br>*"We will work through these decisions together."*
Offer the person some control by letting them indicate what they can cope with discussing.	*"How much information do you want me to share with you about what is likely to be ahead?"*
Talking 'around' the issue rather than addressing it directly raises anxiety.	Name the issue that needs to be discussed. Say *"dying"* rather than *"passing away"*.<br>Be succinct, direct, honest, and gentle.

Table 11: Strategies and suggested language for dealing with sadness and fear

**Sadness and fear**	
"Well, it was like being punched in the stomach really because I'd sort of coped with my kidney problem, and I'd lived with the hope that my kidneys would see me through, even though they were poor." (Mary, person with kidney failure)	
**Key ideas**	**Strategies and suggested language**
Responding to emotion is often necessary to allow a difficult conversation to proceed.	Allow silences so the person can express their feelings.<br>Name the feelings, e.g., fear, regret, worry.<br>Provide non-verbal support, e.g., maintain eye contact, face the person, and keep an open posture.
Tears and other emotional reactions are natural when discussing end of life plans.<br><br>Listening is therapeutic, even if you can't 'fix' the situation.	Ask the person to describe what the tears are about.<br>*"Can you describe what is making you feel sad/upset/scared?"*<br>Explore feelings.<br>*"Tell me more."*<br>Express empathy.<br>*"I am sorry that this is so sad/upsetting/scary for you. This must be so hard to talk about."*<br>Provide support and encouragement.<br>*"I know this is a hard conversation to have, but I think it is important and will help make sure that we have a back-up plan in case we need one."*<br>Obtain permission to proceed.<br>*"Can we talk a bit more about this?"*<br>Express respect for the person's emotional strengths.<br>*"I can see you are a person who feels things strongly and I have a lot of respect for your strength in staying with this hard discussion."*

Sometimes, backing off is a good temporary strategy. Stay calm.	If necessary, offer to take a break and proceed later.<br><br>*"I can see that this is a really tough conversation for you. Would you like to take a break for today and try to talk about it next time?"*
People are often frightened of alienating a health professional by crying – reassurance and staying present can mitigate this.	Reassuring the person before hearing what they are distressed about cuts off communication. Provide targeted reassurance, if possible, after the person has expressed their major concerns.<br><br>*"I wish I could promise you that you will be able to stay at home until the end. What I can say is I will do everything in my power to make that happen. I also want to be honest in telling you that unexpected things can happen that would make it better for you to be in the hospital. We will include you (and your family) as much as possible in this kind of decision."*

Section E: Supporting services and information

Palliative care

Involving palliative care services means that care is shared across different specialities, which may include primary care and community-based nursing teams. While palliative care needs increase in the last few months of a person's life, this service may be accessed alongside standard kidney care management at any point in the kidney treatment pathway. It is helpful to palliative care services if the kidney care team has had difficult conversations with people in advance.

We have a community matron, and she first got involved with us over the two years ago when he was first told he was going to die.
(Louise, carer)

Then it was so lovely, the palliative care team came round and actually, the woman, the doctor, my dad lives in Suffolk, and the consultant all met him. He was totally aware that he was going to end dialysis. And they put in so many services, it was amazing.
(Melissa, carer)

The district nurses came…and one of them told me it was palliative care. I says, 'What does palliative care mean?' She says, 'End of life.' And I told my wife, and we were both absolutely shocked…because I'm not at the end of life. I don't see myself as being at the end of life.
(Henry, person with kidney failure)

Suggesting a discussion about palliative care can shock people if they perceive that this implies they are approaching the end of life. It can help to explain to people that many of the treatments they have been receiving are palliative options, i.e., those that help people cope with the symptoms of their kidney disease and maintain their quality of life. The next step is to involve health professionals with more expertise about which treatments will fit into the person's life in the future.

Ask the person about their understanding of palliative care. Discuss the type of care that might be involved, and emphasise that it is available to anyone who needs it, irrespective of their prognosis or chosen treatment. Recommend next steps that are based on health status, medical options, treatment burden, and a person's own values and preferences, Table 12 (page 27).

And suddenly, in the course of about 30 minutes, the renal consultant and the GP and the hospice talked to each other. It was absolutely stunning how quickly the thing got working. It was remarkably quick, remarkably effective.
(David, bereaved carer)

Table 12: Suggested language for making a recommendation about palliative care options

Suggested language
"Based on the decline in your kidney function and your wishes to stay at home, I recommend that we talk to the local hospice, who can support you to try to stay at home with your family, and make sure that you are as comfortable as possible."

Patient information

Offer people additional information resources if they are available. Patient information can improve knowledge, inform, prepare, and help people make decisions about treatment and care, serve as an aide-memoire to reinforce information provided in a consultation, act as a prompt to stimulate discussions between health professional and patient, and be read at home and shared with family members [21,22, 23,24,25].

Few people we spoke to had received additional written information. Those who did receive information found it useful, kept hold of it, and referred to it post-consultation.

The hospice gave us a couple of background information leaflets when we first contacted them. Those were helpful.

(David, bereaved carer)

Yeah, it's pretty good. There's still a lot of questions to be asked, but I do refer to it. This is the reason that it sits on the side in the kitchen. I do refer to it sometimes when something comes up.

(Jane, person with kidney failure)

In the absence of additional information provided by a health professional, people searched online, and via kidney patient groups and social media.

I just put it in Google and read everything, everything I can find. I'll look at the hospice sites and stuff and see, various hospice sites that have got things on the internet and have a look and see what they say about palliative care and end of life and things.

(Louise, carer)

Page 29 signposts some patient information resources to share with people with kidney failure and their families.

Psychological support

People with kidney failure recognised the importance of additional support to help them come to terms with their illness and treatment. Where appropriate and if it is available within your service, offer a referral to psychology services to discuss any concerns that they may have about their illness (Table 13).

> **And she said, 'Oh, I'm going to make a referral.' I said, 'He's really upset, you know, and I think it's really stressful for him.' And she said, 'I'm going to refer him to counselling. Would he agree to that?' And he said yes.**
>
> **(Melissa, carer)**

> **We need some kind of psychiatric-type help. Not a psychiatrist, but a psychotherapist nurse would help on the ward, basically, to put it bluntly, we're on death row waiting for execution, and so basically to soften the problems that people have.**
>
> **(Henry, person with kidney failure)**

Table 13: Suggested language for making a recommendation for a referral to another service

Suggested language
"In order to help with your very natural concerns about your future, I'd like you to see our psychologist, who is an expert in working with people in your situation."

Carer and bereavement support

Family members talked about the difficulties of looking after someone with kidney failure. They may benefit from access to information about the likely progress of a person's condition, information about the support services available, practical, emotional, and bereavement support [12,19]. Page 29 includes details of resources to share with family members and carers.

> **I mean, at the moment now, we're involved with the hospice in town, and there is chance of me going to a carers' meeting with other carers to discuss. I think that'll help me.**
>
> **(Louise, carer)**

> **They were certainly standing by and said, 'Don't hesitate in any way, shape or form.' You'd just get in touch with them and they would be there for me, so I never felt isolated or alone with it.**
>
> **(Lucy, bereaved carer)**

> **You see, it is always the patient really that gets the attention, and I don't think there's enough understanding or support for carers. As long as you're coping, on the whole, you're left to get on with it. You do feel, feel quite lonely at times.**
>
> **(Rebecca, bereaved carer)**

Section F: Further information

Health professional information and guidance

NHS – advance care statements: www.nhs.uk/conditions/end-of-life-care

NHS – chronic kidney disease: www.nhs.uk/conditions/kidney-disease

NHS – Think Kidneys: www.thinkkidneys.nhs.uk

NICE guidance: www.nice.org.uk/guidance/ng107

Kidney patient charities

Kidney Care UK: www. kidneycareuk.org

Kidney Research UK: www.kidneyresearchuk.org

National Kidney Federation: www.kidney.org.uk

UK Kidney Association – UK Renal Registry: https://ukkidney.org/patients/information-resources

Kidney patient organisations and networks

Kidney Patient Involvement Network: https://kpin.org.uk

UK Kidney Association: https://ukkidney.org

End of life and bereavement charities

Cruse Bereavement Support: www.cruse.org.uk

Hospice UK: www.hospiceuk.org

Sue Ryder Palliative Care: www.sueryder.org/how-we-can-help

Patient decision aids

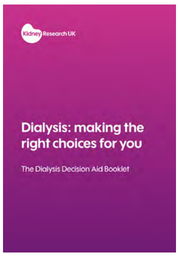

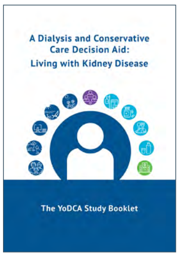

The Diaysis Decision Aid (also known as YoDDA) and Yorkshire Dialysis and Conservative care Decision Aid (YoDCA) are resources developed to support people with kidney failure making difficult treatment decisions.

These resources were developed with unrestricted project grants provided by Kidney Research UK, in partnership with Baxter Healthcare Ltd, and Kidney Research Yorkshire from our team's research since 2004, into people’s experiences of making kidney disease treatment decisions and delivering kidney care. These resources are available online:

Dialysis Decision Aid (YoDDA): https://kidneyresearchuk.org/wp-content/uploads/2019/05/KR-decision-Aid-DOWNLOAD.pdf

Yorkshire Dialysis Decision and Conservative Care Aid (YoDCA): https://www.kidneyresearchyorkshire.org.uk/wp-content/uploads/2020/09/Diaysis-Conservative-Care-FINAL-SEPT-2020.pdf

Space for notes and local information

References

Research used to inform this booklet's content:

1. Hackett J, Bekker H, Bennett MI et al. Developing a complex intervention to support timely engagement with palliative care for patients with advanced cancer in primary and secondary care in the UK: A study protocol. BMJ Open, 2018, 8(5):e022835.

2. Schell J & Cohen R. A communication framework for dialysis decision-making for frail elderly patients. CJASN, 2014; 9(11):2014–2021.

3. British Heart Foundation. Difficult conversations heart failure booklet: Making it easier to talk to people with heart failure about the end of life. National Council for Palliative Care, 2014; ISBN: 978-1-910286-01-2.

4. Mandel EI, Bernacki RE, Susan D, Block SD. Serious illness conversations in ESRD. CJASN, 2017; 12(5):854–863.

5. Davison SN, Levin A, Moss AH et al. Executive summary of the KDIGO Controversies Conference on Supportive Care in Chronic Kidney Disease: Developing a roadmap to improving quality care. KI Reports, 2015; 88:447–459.

6. Senanayake S, Gunawardena N, Palihawadana P et al. Symptom burden in chronic kidney disease: A population based cross sectional study. BMC Nephrol, 2017; 18:228.

7. Al-Mansouri A, Al-Ali FS, Hamad AI et al. Assessment of treatment burden and its impact on quality of life in dialysis-dependent and pre-dialysis chronic kidney disease patients. Res Social Adm Pharm, 2021; 17(11):1937–1944.

8. Steenkampa R, Pyarta R, Fraser S. UK Renal Registry 20th annual report: Chapter 5 Survival and cause of death in UK adult patients on renal replacement therapy in 2016: National and centre-specific analyses. Nephron, 2018; 139(suppl1):117–150.

9. Sturgill D & Bear A. Unique palliative care needs of patients with advanced chronic kidney disease — the scope of the problem and several solutions. Clinical Medicine, 2019; 19(1):26–29.

10. O'Halloran P, Noble H, Norwood K et al. Advance care planning with patients who have end-stage kidney disease: A systematic realist review. J Pain Symptom Manage, 2018; 56(5):795–807.e18.

11. Gomm S, Farrington K, Brown E et al. End of life care in advanced kidney disease: A framework for implementation. NHS Improving Quality, 2015.

12. Marie Curie Policy and Public Affairs. Difficult conversations with dying people and their families, 2014.

13. National Institute for Health and Care Excellence. End of life care for adults. Quality standard, 2011, www.nice.org.uk/guidance/qs13.

14. National Palliative and End of life Care Partnership. Ambitions for palliative and end of life care: A national framework for local action 2015–2020, 2015.

15. Lazenby S, Edwards A, Samuriwo R et al. End of life care decisions for haemodialysis patients – 'We only tend to have that discussion with them when they start deteriorating'. Health Expect, 2017; 20:260–273.

16. de Barbieri I, Strini V, Noble H et al. Nurse-perceived facilitators and barriers to palliative care in patients with kidney disease: A European Delphi survey. J Ren Care, 2022; 48:49–59.

17. Saeed F, Ladwig SA, Epstein RM et al. Dialysis regret: Prevalence and correlates. CJASN, 2020; 15(7):957–963.

18. Stringer S & Baharani J. Why did I start dialysis? A qualitative study on views and expectations from an elderly cohort of patients with end-stage renal failure starting haemodialysis in the United Kingdom. Int Urol Nephrol, 2012; 44(1):295–300.

19. Bekker HL, Winterbottom A, Gavaruzzi T, Mooney A, Wilkie M, Davies S, Crane D, Tupling K, Mathers N of the Yorkshire Dialysis Decision Aid (YoDDA) Team. The Dialysis Decision Aid Booklet: Making The Right Choices for You. Kidney Research UK: Peterborough, UK. (2020). Date of next content review and update, January 2023. (Accessed via www.kidneyresearchuk.org/DialysisDecisionAid).

20. Winterbottom A, Mooney A, Russon L, Hipkiss V, Williams R, Ziegler L, Finderup J, Bekker HL. The Yorkshire Dialysis and Conservative Care Decision Aid Booklet (YoDCA). Kidney Research Yorkshire, West Yorkshire, UK. 2020. Date of next content review and update 2023. (Accessed via kidneyresearchyorkshire.org.uk).

21. Winterbottom A, Conner M, Mooney A et al. Evaluating the quality of patient leaflets about renal replacement therapy across UK renal units. Nephrol Dial Transplant, 2007; 22(8):2291–2296.

22. Winterbottom A, Stoves J, Ahmed A et al. Patient information about living donor kidney transplantation across UK renal units: A critical review. J Ren Care, 2021; 1–11.

23. Winterbottom AE, Mooney A, Russon L et al. Critical review of leaflets about conservative management used in UK renal services. J Ren Care, 2020; 46(4):250–257.

24. Winterbottom AE, Mooney A, Russon L et al. Kidney disease pathways, options and decisions: an environmental scan of international patient decision aids, Nephrology Dialysis Transplantation, 2020; 35(12):2072–2082.

25. Winterbottom AE, Gavaruzzi T, Mooney A et al. Patient acceptability of the Yorkshire Dialysis Decision Aid (YoDDA) booklet: A prospective non-randomized comparison study across 6 predialysis services. Perit Dial Int, 2016; 36(4):374–381.

Study details

Difficult conversations study team

The study team thank the people with kidney failure and their family members who took part in interviews, the patients, PPI team, academics, and health professionals who commented on drafts of the booklet, and the kidney professionals working in Leeds and Manchester kidney units, the Kidney Patient Information Network (KPIN), and the National Kidney Federation who supported study recruitment.

Study team members

Dr Anna Winterbottom (Health Services Researcher, Leeds Teaching Hospitals NHS Trust, University of Leeds)

Dr Andrew Mooney (Consultant Renal Physician, Leeds Teaching Hospitals NHS Trust)

Dr Helen Hurst (Consultant Nurse, Central Manchester University Hospitals NHS Foundation Trust)

Dr Lynne Russon (Palliative Care Consultant, Wheatfields Hospice, Leeds)

Prof Paula Ormandy (Professor in Long Term Conditions Research, University of Salford)

Independent advisory team members

Prof Fliss Murtagh (Professor in Palliative Medicine, Hull York Medical School, University of Hull)

Prof Hilary Bekker (Professor of Medical Decision Making, University of Leeds)

Dr Barny Hole (Kidney doctor in training, North Bristol NHS Foundation Trust, University of Bristol)

Dr Emma Murphy (Associate Professor in Nursing, University Hospital Coventry, Warwickshire NHS Trust)

Ian Simkin (Patient representative, kidney transplant recipient)

Patient and public involvement (PPI) advisory team members

Keith Bucknall (PPI chair; kidney transplant recipient)

Jan Cooper (Kidney transplant recipient)

Richard Williams (Kidney transplant recipient)

Conflicts of interest

The authors have no conflicts of interest, financial or otherwise. The funders, authors, and their employers do not stand to benefit from changes in practice made after reading this booklet.

Project funding

Our project 'Difficult Conversations Booklet: A resource for health professionals to help them talk to people with kidney failure about palliative care issues' was funded by Kidney Care UK and the British Renal Society (now UK Kidney Association; ref 19-001; 2021-2022).

Collaborating organisations

The Association of Nephrology Nurses (ANN UK) endorse the use of this booklet.

Contact

Anna Winterbottom (anna@winterbottom.co.uk), Leeds Renal Unit, St James's University Hospital, Leeds, UK.

If you wish to undertake a research or quality improvement project using the booklet, contact Dr Winterbottom about collaboration.

Referencing this booklet

Winterbottom A, Mooney A, Hurst H, Russon L, Ormandy P, Bucknall K, Murtagh F, Bekker HL, Hole B, Murphy E, Simkin I. Difficult conversations: Talking with people about kidney failure, end of life and advance care planning: A guide for health professionals. (2022). Kidney Care UK & the British Renal Society (now UK Kidney Association). Accessed via: https://www.kidneycareuk.org/health-professionals/difficult-conversations

© Leeds Teaching Hospitals NHS Trust (November, 2022, Winterbottom & Mooney, Leeds Renal Unit)

Kidney, geriatric, allied, and palliative care health professionals, and people with kidney failure reviewed various iterations of this booklet to ensure that its content is accurate, complete, and relevant.

More copies of this booklet are available from: https://www.kidneycareuk.org/health-professionals/difficult-conversations/

© Leeds Teaching Hospitals NHS Trust, Leeds, UK (2022)

Authors: Yorkshire Dialysis and Conservative Care Decision Aid (YoDCA) study team:
Winterbottom A, Mooney A, Hurst H, Russon L, Ormandy P, Bucknall K,
Murtagh F, Bekker HL, Hole B, Murphy E, Simkin I.

Produced by: Medical Illustration Services • MID code: 20220628_004/NR

LTH4427 1st edition (Ver 1)
Publication date: 11/2022
Review date: 01/2025
